# Combined effects of cotyledon excision and nursery fertilization on root growth, nutrient status and outplanting performance of *Quercus variabilis* container seedlings

**DOI:** 10.1371/journal.pone.0177002

**Published:** 2017-05-18

**Authors:** Wenhui Shi, Mark Bloomberg, Guolei Li, Shuchai Su, Liming Jia

**Affiliations:** 1 Key Laboratory for Silviculture and Conservation, Ministry of Education, Beijing Forestry University, Beijing, China; 2 Beijing Laboratory of Urban and Rural Ecological Environment, Beijing, China; 3 Department of Land Management and Systems, Faculty of Agribusiness and Commerce, Lincoln University, Christchurch, New Zealand; Austrian Federal Research Centre for Forests BFW, AUSTRIA

## Abstract

Artificial excision of the distal part of acorns in order to promote germination is well researched in oak seedling cultivation studies. However, studies of combined effects of cotyledon excision and nursery fertilization on container seedlings are lacking, especially for seedling root growth and outplanting performance. This study aimed to explore the main effects of cotyledon excision on *Quercus variabilis* seedling emergence characteristics and demonstrated the combined effects of cotyledon excision and nursery fertilization on seedling quality to improve *Quercus variabilis* seedling outplanting performance. Four cotyledon excision treatments and two classes of nursery fertilization were implemented. Seedling emergence was noted every week after sowing. Seedling dry mass, morphology, and nutrient status were assessed at the end of the nursery season. After the first outplanting season, the aforementioned measurements along with seedling survival were determined once again. The results showed that cotyledon excision generally induced greater and more rapid seedling emergence, but did not affect shoot emergence synchronicity. The highest total emergence and emergence rate occurred with Intermediate excision (1/2 of the distal end of acorn was excised). Effects of nutrient loss due to cotyledon excision on seedling quality and outplanting performance were somewhat compensated by nursery fertilization. Nursery fertilization promoted dry mass increment (the net increment from *T*_*0*_ to *T*_*2*_ for dry mass) for excised seedlings after outplanting, resulting in better performance for Slight (1/3 of the distal end of acorn was excised) and Intermediate excision treatments in the field. Thus we conclude Intermediate excision combined with reasonable nursery fertilization can be recommended for production of nursery grown seedlings for afforestation.

## Introduction

Oak (*Quercus*) species are widely distributed in the northern hemisphere [1]. Matured acorns feature large size, and are easily found by animals such as rodents, rabbits and birds [2], which results in preferential dispersal for acorns and high returns for predators [3]. In nature, the embryo parts of acorns commonly escape from being eaten due to the bitter and astringent taste [4], and have the potential to develop into seedlings [5–11]. In this way, acorns achieve both dispersal and plant regeneration, paying a price of acorn damage by the seed predators [12–16]. Thus, establishing oak species will improve the habitat function of sites by providing mast for wildlife due to their common characteristic of large partially-edible seeds.

Although acorn dispersal is facilitated by predators, poor germinability can restrict acorn germination and seedling performance [17–19]. Acorn germination rates are usually low and seedling emergence is unsynchronised, so that the duration of germination can last for more than a few weeks [20]. The asynchronous germination results in uneven development of seedlings. Earlier germinated plants may overshadow neighboring seedlings and restrict access to water in the process of container seedling production [21]. In the last decades, there are many studies focused on these problems that have used a series of pre-germination techniques, such as stratification or exposure of seeds to cold or warm temperatures for variable time periods [22,23]. These techniques normally increased acorn emergence but did not promote germination synchronicity. ISTA (International Seed Testing Association) [24] reported that cutting off the distal end of acorns helped to enable faster and more uniform germination in germination tests. Branco et al. [6] and Giertych and Suszka [9] found that acorn damage at the distal end by seed predators could induce faster and more synchronous germination in *Quercus robur* L. seedlings—possibly due to increased indoleacetic acid levels [25,26] or faster imbibition through the damaged seed coat [27]. Accordingly, excising the distal ends of acorns was considered as a suitable practice to accelerate germination in container nurseries [9,11,20]. Forest managers have imitated this by excising distal ends of acorns before sowing them into soil to improve seedling emergence [28].

However, acorn excision will result in loss of cotyledon nutritional reserves and may therefore reduce early seedling growth [29]. Partial cotyledon loss might also impact root system development in the nursery and therefore further nutrient absorption from the soil [9,11]. Thus seedling growth may be restricted when outplanted into field. The relationship of field-sown seedling growth with the extent of simulated acorn damage has been well documented [6,8,10,20,21,30,31]. But there is still a lack of information on effects of cotyledon excision on container nursery seedlings, from the outplanting performance perspective. Furthermore, acorns in nature may be dispersed into various sites with different nutrient supply capacities, and different oak species may respond inconsistently to acorn damage intensities in different soil fertility conditions. *Quercus variabilis* Blume, a deciduous native tree with straight and dominant taproots, is an economically and ecologically valuable species that is widely planted in China. Published papers have described seed provenance, seedling growth and growth response to nursery practices including fertilization and mycorrhizal inoculation [10,32–34]. In the current study, we hypothesized that cotyledon excision enabled better emergence characteristics of *Quercus variabilis* seedlings and the nutrient loss due to artificial cotyledon excision might be compensated by sufficient soil nutrients. Therefore, we aimed to reveal the effects of different cotyledon excision intensities on emergence characteristics, and we also aimed to explore the combined effects of partial cotyledon excision and nursery fertilization on *Quercus variabilis* container seedling growth and outplanting performance. In order to achieve our object, we compared the emergence characteristics between different cotyledon excision intensities, and investigated the seedling root growth. Finally, we comprehensively evaluated the effects of cotyledon excision on container-grown seedling cultivation, from the viewpoints of seedling emergence, seedling growth at nursery and outplanting performance.

## Material and methods

Acorns for this study were obtained from five parent trees in mid-September from Sizuolou Forest Farm in Pinggu, Beijing, China (117°148′E, 40°282′N; elevation 316–467 m). Mature acorns were collected in autumn 2011 and preprocessed as detailed in Li et al. [34]. The average dry mass of acorns was 3.49 g, and the N, P, K content per acorn was 23.7 mg, 3.2 mg and 28.8 mg, respectively.

### Nursery phase

**Fig 1:**
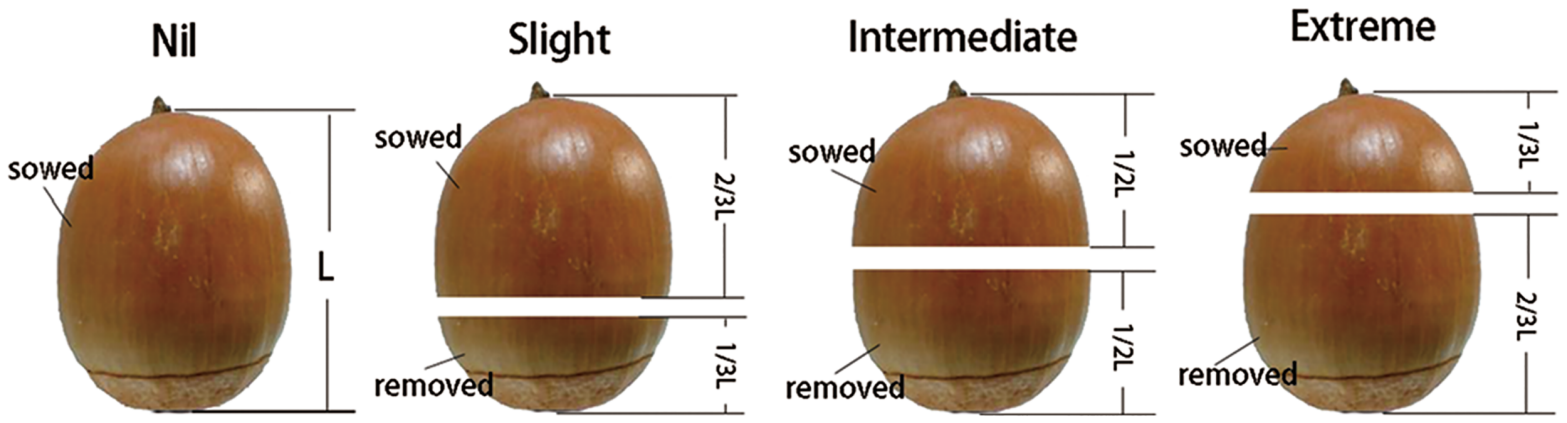
Four categories of *Quercus variabilis* acorns after artificial cotyledon excision before sowing. Nil: intact control, Slight: cutting off 1/3 of the distal end of acorns, Intermediate: cutting off 1/2 of the distal end of acorns, Extreme: cutting off 2/3 of the distal end of acorns.

After about 5 months’ storage, acorns with similar size and fresh weight were randomly assigned to one of four excision (E) treatments: Nil—intact control, Slight—cutting off 1/3 of the distal end of acorns, Intermediate—cutting off 1/2 of the distal end of acorns, Extreme—cutting off 2/3 of the distal end of acorns (Fig 1).

To ensure a sufficient number of seedlings would be available for sampling and planting, we sowed 1800 acorns (450 per treatment). Each individual acorn was sown at a depth of 1–2 cm in a plastic container (volume 1050 ml, caliber 100 mm, bottom-diameter 60 mm and depth 200 mm, Guangzhou Jin Ying Fat Garden Products Co., Ltd) filled with a mixture of 3:1 (v:v) peat (Pindstrup Seeding, pH 6.0, Screening 0–6 mm): perlite (5 mm diameter, Xinyang Jinhualan Mining Co., Henan, China) on 24^th^ February 2012. Thirty containers were randomly assigned to each of 60 trays (53.0 cm long × 44.5 cm wide); thus, we initially employed 15 trays for each excision treatment. Trays were labeled and randomly placed in a greenhouse with minimum—maximum temperatures of 21–28°C, 40–60% relative humidity and natural daylight (minimum and maximum light flux: 200 and 1,000 μmol m^−2^ s^−1^, respectively) at the Chinese Academy of Forestry Sciences in Beijing (40°40′N, 116°14′E). Seedling emergence was noted every week for 13 weeks from the date of sowing.

The trays of each excision treatment (ignoring the 5 blocks located on the edge of the seedbed) were allocated into two groups (5 trays for each), with one group receiving nil fertilizer in the nursery phase (Low NF), and the other group (High NF) supplied a total of 100 mg N, 40 mg K_2_O, 60 mg P_2_O_5_, 4.5 mg EDTA-2N and 1.4 mg DTPA per seedling for 15 weeks commencing 26^th^ March. The High NF pots were carefully irrigated with 20 ml of fertigation solution once a week and meanwhile the Low NF pots were irrigated with 20 ml water. In the fertilizing process, N was supplied as urea (Xilong Chemical Co., China), an N source that is commonly used in nursery cultivation in China and P, K were supplied as KH_2_PO_4_ (Guangdong Guanghua Sci-Tech Co., Ltd. China). All seedlings were watered to field capacity, approximately two times each week.

After the first growing season when all leaves had fallen (13^th^ November, 2012) (*T*_*0*_), forty seedlings per treatment (eight seedlings per replicated tray) were randomly sampled for morphology and nutrient status analyses. Seedlings were washed gently free of growing medium and then separated into stems and roots. Seedling roots were scanned (Epson Expression 1640XL, Canada) and then analyzed by an image analysis system (WinRHIZO, Regent Instruments Canada Inc.) to determine the root surface area and number of first order lateral roots (FOLR, D>1 mm) based on root diameter. After root scanning, each plant organ type (stems or roots) was oven-dried at 70°C for 48 h to determine its dry mass. The organ samples were subsequently combined to a composite sample for each organ × each individual tray, and ground, sieved through a 0.25 mm screen, then wet-digested using the H_2_SO_4_-H_2_O_2_ method. A standard Kjeldahl digestion with water distillation was used to measure total N by a distillation unit (UDK-152, VelpScientifica, Italy). Phosphorus (P) concentration was determined with a UV-visible spectrophotometer (Agilent 8453, USA) and potassium (K) concentration was quantified with an atomic emission photometry (SpectrAA 220 Atomic Absorption Spectrometer, VARIAN, USA).

The remaining seedlings were removed from the greenhouse and stored outdoors under snow cover during winter until March in the following year.

### Outplanting phase

In early May of 2013, seedlings from the eight nursery treatments were shipped and planted on previously cultivated land in the Northern Experimental Base of Beijing Forestry University at Pingquan, Hebei province (118°40′E and 41°13′N). The site had average elevation of 765 m with slope < 2%. The depth of soil varied between 45 and 60 cm. The surface soil (0–20 cm) was a sandy clay loam (73% sand, 11% silt, and 16% clay) with a pH of 6.2 and soil organic carbon of 0.7%. Average total N, available P, and available K were 628.7, 139.5, and 113.5 mg kg^-1^ respectively (S1 Table). The Pingquan area has a temperate continental monsoon climate characterized by dry winter and spring seasons. During 2013, mean annual air temperature was 7.2°C and annual rainfall was 509.6 mm. The soil was ploughed before outplanting. Weeds were removed by hand.

The field experiment was arranged as a randomized complete block experimental design with five replicates. Fifteen randomly selected seedlings from one tray per nursery treatment were planted in single parallel rows within each replicate and each block was separated from adjacent blocks by 2 m buffers. A total number of 600 seedlings were planted. Height (H) (root-collar to the tip of the terminal bud) and root-collar diameter (RCD) were measured immediately after planting (*T*_*1*_) and in early November of the year (*T*_*2*_), when growth ceased on this site. In late August, survival was calculated as number of seedlings of the original fifteen remaining alive for each nursery treatment replicate. Fifteen seedlings were randomly sampled in early November (*T*_*2*_) from each treatment (3 per replicate parallel row) for dry mass (*W*) measurement. Net increment from *T*_*0*_ to *T*_*2*_ for dry mass was calculated with the following equation.

$$Net\;increment\;=W_{2}-\;W_{0}$$ (1)

### Statistical analyses

All statistical analyses were performed using IBM 18.0 for Windows (SPSS Statistic, Inc., Chicago, IL, USA).

#### Seedling emergence

We used a Chi-square test to recognize differences in the maximum cumulative proportion (%) of seedling emergence (hereafter total emergence) through the experiment period between experimental excision treatments. Cumulative emergence versus time for each treatment was modelled using a Logistic linear regression to graph the emergence trends. The form of the regression model was:

$$Y_{x}=Y_{0}+\frac{a}{1+{\left(\frac{x}{b}\right)}^{c}}$$ (2)

where *Y*_*x*_ is the cumulative proportion (%) of seedling emergence at time *x* (days) after sowing, *Y*_*0*_ the asymptotic value for the model, *a*, *b* and *c* the shape parameters.

**Fig 2:**
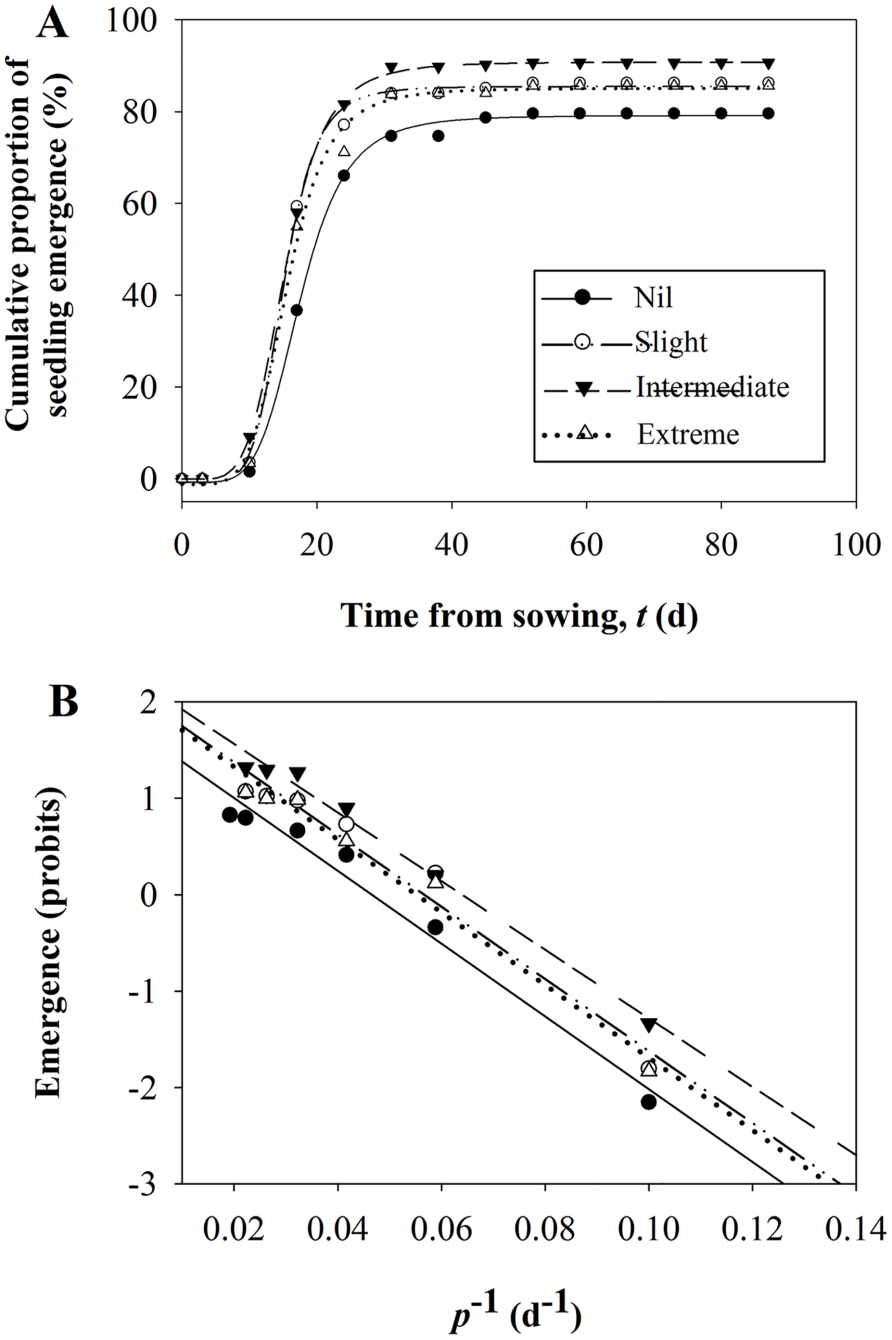
Seedling emergence characteristics for all provenances combined for each of the experimental treatment. Logistic regression model (A) for cumulative emergence of *Quercus variabilis* container seedlings in relation to time from sowing for the four cotyledon excision treatments (Nil, Slight, Intermediate and Extreme) and transforming the proportion of acorns that emergence to probit values in a linear relationship with *p*^-1^ (B). Both calculative proportion of emergence and probit values were plotted as the means of fifteen replications for each excision treatment, and 30 acorns per replication.

The cumulative emergence curve for a population of non-dormant seeds is often sigmoidal, but not symmetrical, since the rate of increase in cumulative emergence slows markedly as it approaches the maximum (Fig 2A). This asymmetry can be accommodated by plotting the emergence % against the reciprocal of the period of time from sowing, enabling the fitting of a cumulative normal distribution curve to the emergence.

Where the explanatory variable (reciprocal of time to emergence) is assumed to follow a normal distribution, then a probit analysis approach [35] can be used (Fig 2B). We used a probit analysis with the general linear model expressed as:

$$g=\Phi (\gamma )=\Phi (\beta_{0}-\beta_{1}p^{-1})$$ (3)

where *g* is emergence as a proportion of acorns sown; *γ* is the linear predictor (here emergence in probits); *β*_0_ is the maximum emergence in probits; *β*_1_ describes the rate of decrease in probit emergence as *p*^-1^ increases, and *p*^-1^ refers to the reciprocal of the period of time from sowing, or “emergence rate” (*ER*).

From this model were estimated, 1) the median emergence rate (*ER*_50%_), calculated as the intercept of emergence in probits against *p*^-1^ Eq (4a); 2) the value of standard deviation of the emergence rate (*SD*) Eq (4b). *SD* scales with emergence synchrony, with lower values indicating more synchronized emergence. The calculation process can be described as [35]:

$$ER_{50\%}=\frac{\beta_{0}-\gamma }{\beta_{1}},\;with\;\gamma =0,\;so\;that\;ER_{50\%}=\frac{\beta_{0}}{\beta_{1}}$$ (4a)

$$SD=\frac{1}{\beta_{0}}$$ (4b)

The calculated results of *ER*_50%_ and *SD* for each replicate for the four excision treatments were then analyzed using one-way ANOVA, to identify differences in emergence rate and synchrony between treatments.

#### Seedling dry mass, morphology, nutrition and growth

Two-way ANOVA was used to analyze the effects of excision, nursery fertilization and their interactions on seedling dry mass, and morphological and nutritional attributes after the first (nursery phase, *T*_*0*_) growing season, and on the net increment of seedling dry mass at the end of the second (outplanting, *T*_*2*_) growing season. Separation of means for the measurements were ranked according to the Duncan test at α = 0.05. The “explore” function of SPSS was used to examine data prior to the ANOVA to ensure that normality and variance homogeneity requirements were met and that no transformations were necessary.

### Ethics statement

All necessary permits were obtained for the described laboratory, nursery and field experiments. The laboratory experiment was approved by the Beijing Forestry University, Beijing, China; the nursery experiment was approved by the Chinese Academy of Forestry Sciences, Beijing, China; and the field experiment was approved on this site by the Northern Experimental Base of Beijing Forestry University, Pingquan, Hebei, China. The subject species in our study (*Q*. *variabilis*) is a deciduous native tree species that is widely planted in China. We confirm that the field studies did not involve endangered or protected species.

## Results

### Seedling emergence

Cotyledon excision improved the total emergence of *Q*. *variabilis* seedlings (Fig 2A, S2 Table). Emergence for the three excision treatments ranged from 85% to 91% and for the non-excised acorns was 80%, which was significantly lower than for the excision treatments (Table 1). Greatest emergence occurred in the Intermediate excision rather than either Slight or Extreme excision treatments. The speed of emergence as measured by median emergence rate or rate to 50% emergence (*ER*_50%_) was significantly affected by excision treatments (*p*<0.001). Slight, Intermediate and Extreme treatments had significantly higher *ER*_50%_ than Nil excision. Intermediate excision had the highest *ER*_*50%*_ compared with Slight and Extreme excision, although there was no significant difference between Intermediate and Slight excision. In contrast, excision treatments did not significantly affect the standard deviation (*SD*) of the emergence rate (*p* = 0.792), suggesting that although excision treatments did influence speed of emergence and total seedling emergence, they did not influence emergence synchrony (Table 2).

**Table 1:** *χ*^*2*^ and *P* values from the chi-square test of acorn emergences (multiple comparisons).

Value	Nil- Slight ^a^	Nil- Intermediate	Nil- Extreme	Slight- Intermediate	Slight- Extreme	Intermediate- Extreme
***χ2***	7.051	21.92	5.624	4.394	0.083	5.611
***p***	0.008	<0.001	0.018	0.037	0.774	0.018

^a^ Nil-Slight listed in Table 1 refers to the chi-square test between the experimental treatments of Nil excision and Slight excision. The same manner for Nil-Intermediate, Nil-Extreme, Slight-Intermediate, Slight-Extreme and Intermediate-Extreme.

**Table 2:** The effects of excision treatments on seedling emergence. Statistically analyzed results (mean ± SE) of median germination rate for seedling emergence (ER_50%_) and the value of standard deviation of the emergence rate (SD), using a one-way ANOVA approach. ^a^ Row values not followed by the same letter are significantly different (p < 0.05) according to Duncan’s test.

Value	Nil	Slight	Intermediate	Extreme
***ER***_**50%**_^a^	0.049±0.0009c	0.061±0.0016ab	0.065±0.0014a	0.058±0.0013b
***SD*** ^a^	-0.0281±0.0008a	-0.0279±0.0023a	-0.0262±0.0009a	-0.0272±0.0013a

### Seedling morphological attributes and nutrient attributes at the nursery

In general, fertilization treatment (High NF) increased seedling dry mass, sturdiness and root development compared with the control (Low NF), although effects were not always statistically significant (Table 3). In contrast, effects of excision treatments on seedling size, sturdiness and root development were variable, depending on the organ and attribute being measured.

**Table 3:** *P* values derived from the ANOVA for effects of nursery fertilization (NF), excision treatments (E), and their interaction (NF×E) on *Quercus variabilis* seedling morphological attributes at nursery phase. Seedling RCD, ratio of height to diameter (H/D), root dry mass, stem dry mass, total dry mass, root surface area and the number of first order lateral roots with diameter larger than 1 mm at the taproot junction (FOLR (D>1 mm)) were investigated and analyzed.

Source	RCD	H/D	Root dry mass	Stem dry mass	Total dry mass	Root surface area	FOLR (D>1 mm)
**NF**	<0.001		<0.001		<0.001		0.001
**E**	0.199		0.002		<0.001		0.016
**NF × E**	0.405	0.011	0.173	0.010	0.143	0.039	0.107

Effects of fertilization treatment on seedling nutrient concentrations also varied with the nutrients (N, P or K) and organs (stem or root) being analyzed. Again in contrast, effects of excision treatments on seedling nutrient concentrations were mostly negligible and with one exception, were not statistically significant.

#### Stem RCD and H/D

Treatment effects on seedling sturdiness, as measured by root collar diameter (RCD) and stem height to diameter ratio (H/D), were generally small.

**Fig 3:**
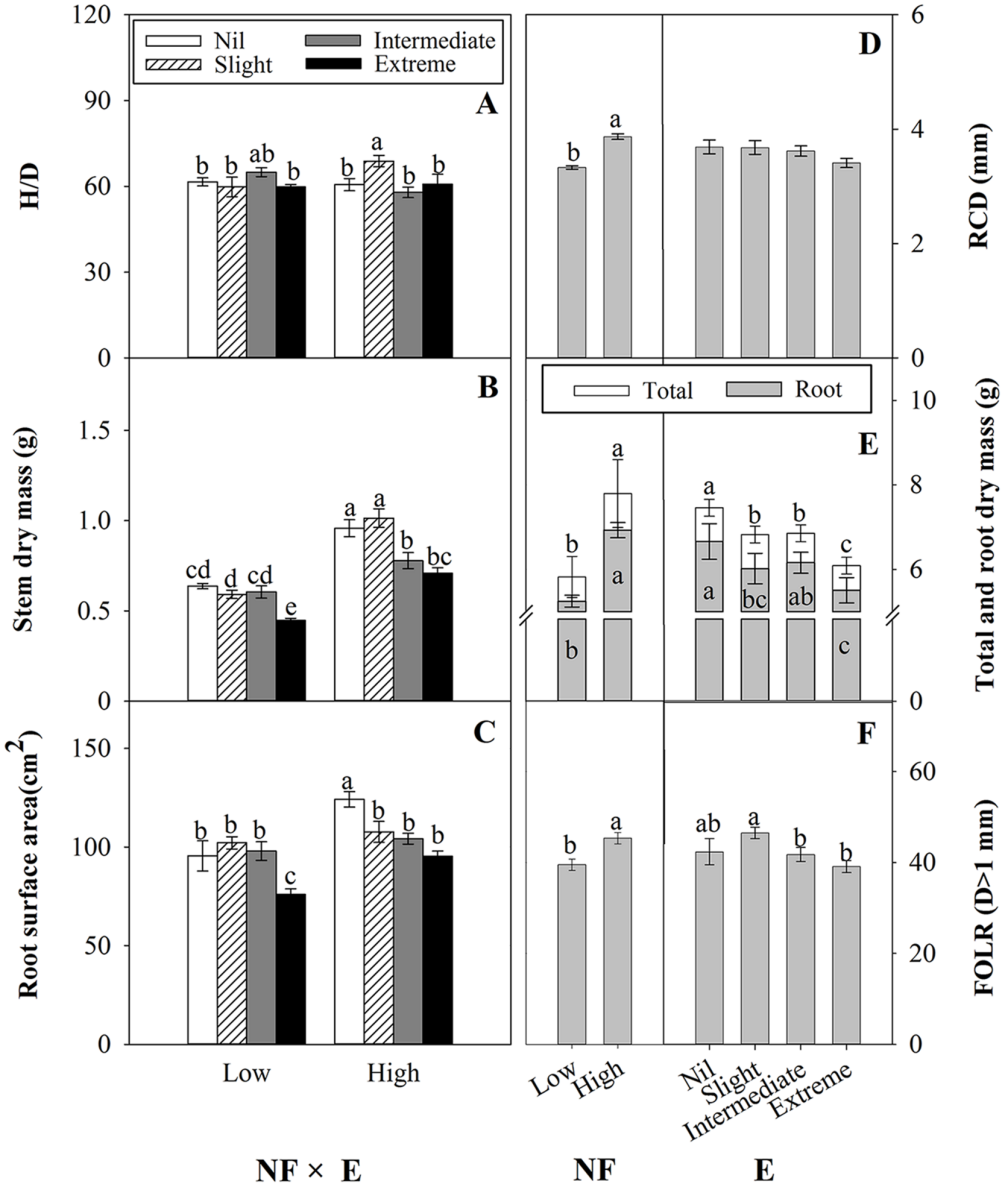
The effects of experimental treatments on plant morphological growth. The left hand side indicates the interaction of nursery fertilization (Low and High) and excision treatments (Nil, Slight, Intermediate, and Extreme) on H/D (A), stem dry mass (B), and root surface (C) (mean ± SE) of *Quercus variabilis* container seedlings after the first growing season. The right hand side indicate the main effects of nursery fertilization and excision treatments on RCD (D), root dry mass and total dry mass (E), and FOLR (D>1 mm) (F) (mean ± SE) of *Quercus variabilis* container seedlings after the first growing season. Bars marked with different letters differ statistically according to Duncan`s test α = 0.05.

Fertilization promoted RCD compared with the control (*p*<0.001, Fig 3D). However, effects of fertilization treatments on H/D were small and almost all were not statistically significant (Fig 3A). Effects of excision treatments on seedling RCD and H/D were also generally small. For RCD, no significant difference between excision treatments was found (*p* = 0.199, Fig 3D) and the interaction term NF×E was also non-significant (*p* = 0.405). For H/D, there was a slight increase with Intermediate excision in the low NF treatment but it was not significantly different compared with other excision treatments. In the High NF regime, Slight excision had significantly higher H/D than all the other treatments, but the size of the effect was not large (Fig 3A).

#### Seedling dry mass and root morphology

Fertilization promoted seedling dry mass and increasing severity of excision treatments had negative effects on seedling dry mass (Fig 3B&3E). The interaction between NF×E significantly affected stem dry mass (*p* = 0.01) but not root and total dry mass (*p* = 0.173 and 0.143, respectively).

Generally, fertilization increased both root surface area (Fig 3C) and first order lateral roots (FOLR) (Fig 3F). However, root surface area was significantly influenced by the interaction between NF×E (*p* = 0.039). For the Low NF, root surface area for the Extreme excision treatment was significantly lower than all other excision treatments. However, for High NF, Slight, Intermediate and Extreme excision all had similar root surface areas which were significantly lower than Nil excision.

In contrast to root surface area, the interaction between NF×E did not significantly affect number of first-order lateral roots (FOLR) (*p* = 0.107). High NF promoted FOLR compared with Low NF, but effects of excision treatments on FOLR were not large.

#### Nutrient attributes

**Fig 4:**
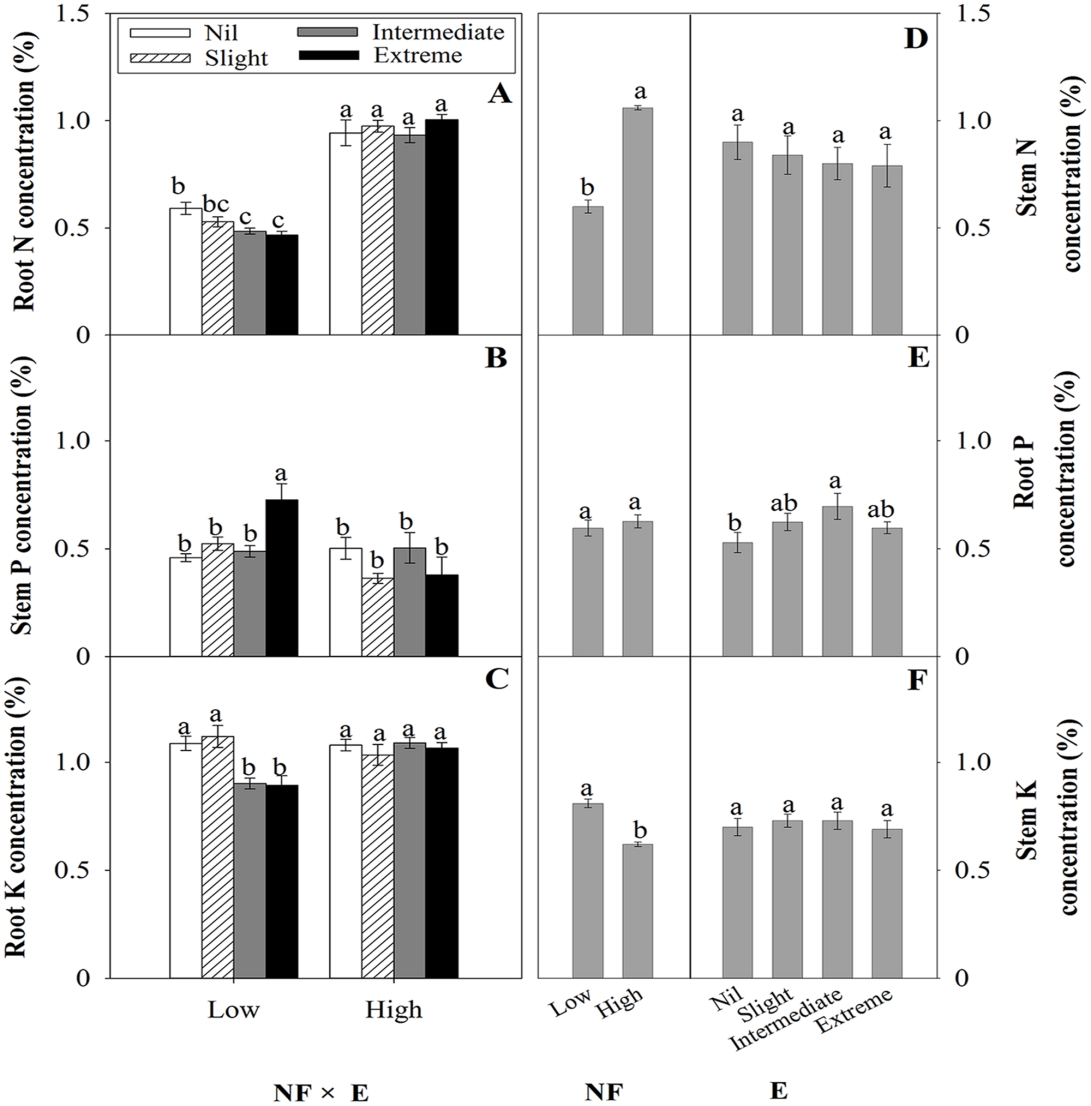
The effects of experimental treatments on plant nutrient concentrations. The left hand side indicates the interaction of nursery fertilization (Low and High) and excision treatments (Nil, Slight, Intermediate, and Extreme) on root N concentration (A), stem P concentration (B) and root K concentration (C) (mean ± SE) of *Quercus variabilis* container seedlings after the first growing season. The right hand side indicate the main effects of nursery fertilization and excision treatments on stem N concentration (D), root P concentration (E) and stem K concentration (F) (mean ± SE) of *Quercus variabilis* container seedlings. Bars marked with different letters differ statistically within each main effect according to Duncan`s test α = 0.05.

Effects of treatments on seedling nutrient concentrations were inconsistent (Table 4, Fig 4). For some treatments, fertilization effects were modified by interactions with excision treatments.

**Table 4:** *P* values derived from the ANOVA for effects of nursery fertilization (NF), excision treatments (E), and their interaction (NF×E) on *Quercus variabilis* container seedling concentration of nitrogen (N), phosphorus (P), and potassium (K).

Source	Nutrient concentration					
	N		P		K	
	Root	Stem	Root	Stem	Root	Stem
**NF**		<0.001	0.487			<0.001
**E**		0.099	0.081			0.326
**NF × E**	0.05	0.889	0.184	0.003	0.001	0.944

For N, high NF generally promoted concentration levels for both stem and root (Fig 4A&4D). For root N levels, high NF also eliminated effects of excision treatments that were significant for the low NF treatments (*p* = 0.050). For P, high NF had no significant effect on root concentration, and little effect on stem concentrations (apart from decreasing stem P concentrations for the Extreme excision treatment) (Fig 4B&4E). Whereas for K, high NF had variable effects, significantly reducing stem K concentration for all excision treatments but increasing root K concentration for Intermediate and Extreme excision treatments, compared with low NF treatments (Fig 4C&4F).

Where excision treatments did not interact with NF treatments (N and K concentrations in stems and P concentration in roots), effects of excision treatments on nutrient concentration were small and with one exception, not significant.

### Outplanting performance

#### Seedling survival

**Fig 5:**
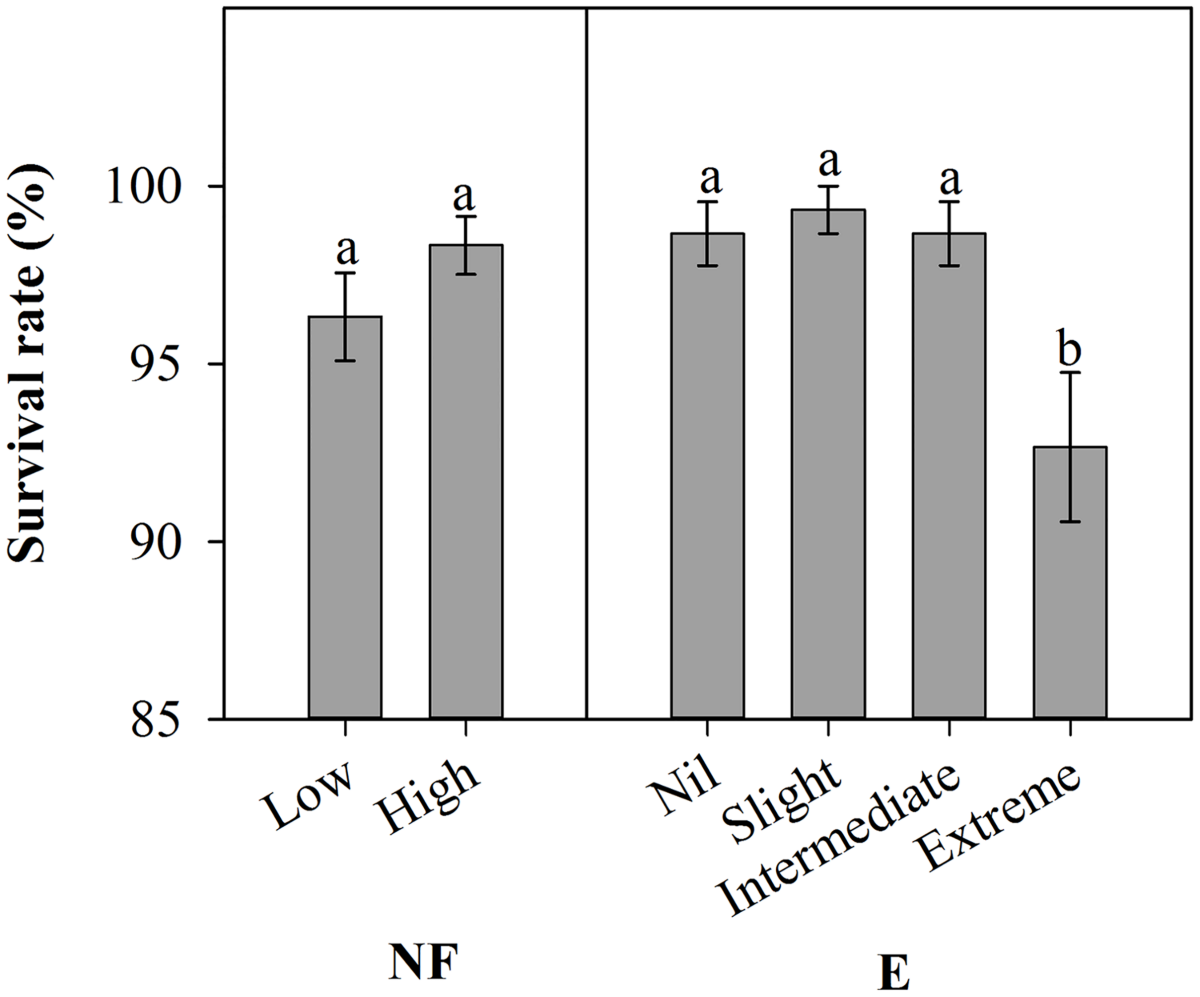
Main effects of nursery fertilization (NF) and excision treatments (E) on seedling survival (mean ± SE) after outplanting. Bars marked with different letters differ statistically according to Duncan`s test α = 0.05.

Fertilization had a small but not significant effect on seedling survival (*p* = 0.099) at the end of outplanting season (Table 5). Even Low NF seedlings exhibited survival percentage as high as 96% (Fig 5). However, excision treatments significantly affected survival (p<0.001). Across both NF treatments, Extreme excision resulted in significantly lower survival than all other treatments.

**Table 5:** *P* values derived from the ANOVA for effects of nursery fertilization (NF), excision treatments (E), and their interaction (NF×E) on *Quercus variabilis* outplanting performance.

Source	Survival	Net increment		
		Stem dry mass	Root dry mass	Total dry mass
**NF**	0.099			
**E**	<0.001			
**NF × E**	0.101	<0.001	<0.001	<0.001

Seedling survival and net increment of stem dry mass, root dry mass and total dry mass were investigated and analyzed.

#### Seedling dry mass increment

Treatment effects were correlated for total seedling dry mass increment and root dry mass increment, because the majority of seedling total dry mass was root dry mass. For the same reason, treatment effects on stem dry mass increment did not correlate as closely with effects on total seedling dry mass increment and root dry mass increment.

**Fig 6:**
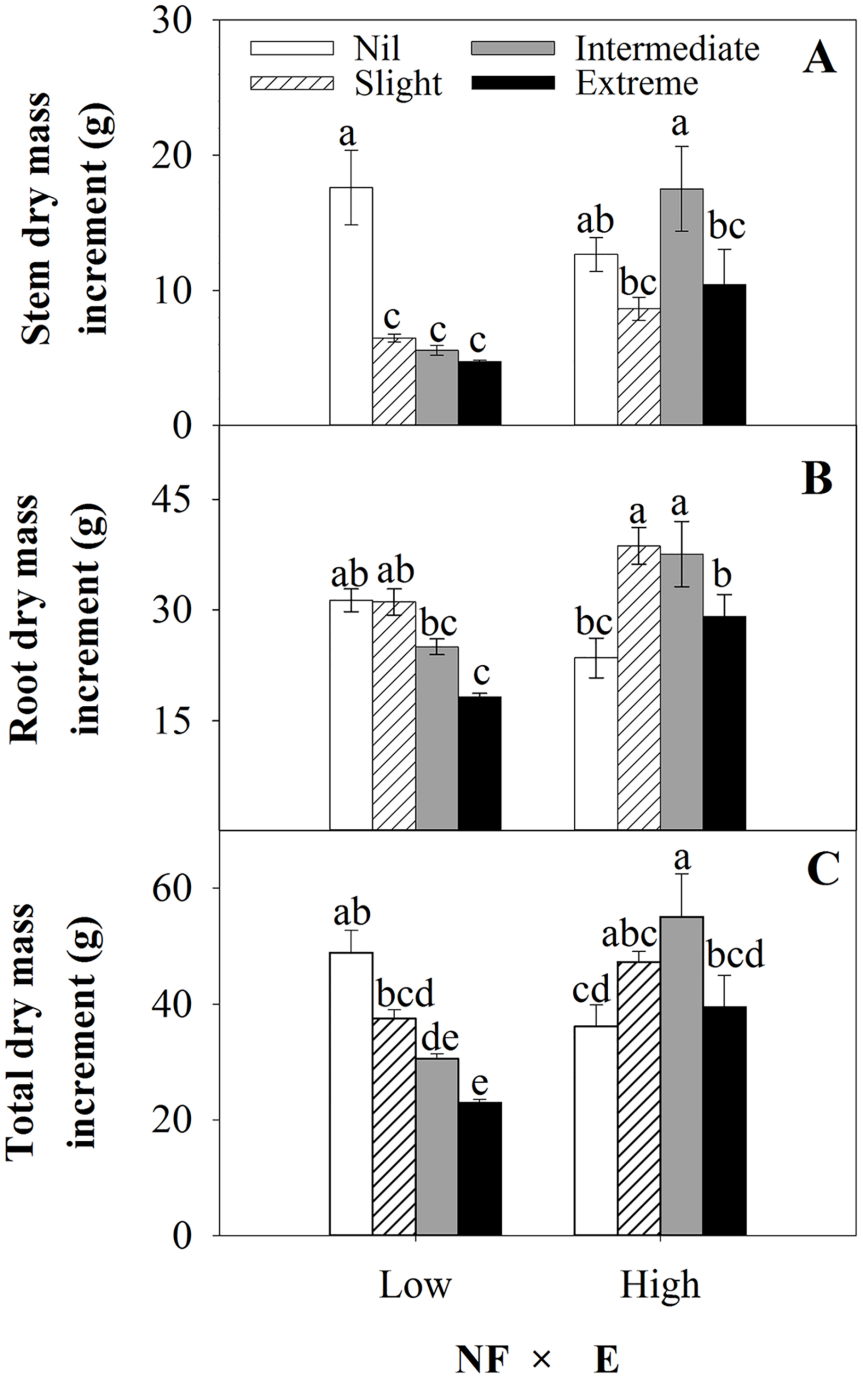
Effects of experimental treatments on seedling dry mass increment after outplanting. The interactions of nursery fertilization (Low and High) and excision treatments (Nil, Slight, Intermediate, and Extreme) on net increment of stem dry mass (A), root dry mass (B) and total dry mass (C) (mean ± SE) of *Quercus variabilis* container seedlings were determined. Net increment is for nursery and outplanting seasons i.e. *Y*_*2*_*-Y*_*0*_. Bars marked with different letters differ statistically according to Duncan`s test α = 0.05.

Generally, High NF promoted dry mass increments for Slight, Intermediate and Extreme excision treatments compared with Low NF, but unexpectedly reduced total dry mass increment for Nil excision (Fig 6C). Excision treatments (E) had more variable effects on stem, root and total dry mass increment (Table 5, Fig 6), and the interaction between NF × E significantly affected dry mass increments for all organs (*p*<0.001 for all). For Low NF, stem, root and total dry mass increments declined with increasing severity of excision. For High NF, excision treatments had no significant effect on stem dry mass compared with Nil excision, however both Slight and Intermediate excision generally promoted root and total dry mass increment compared with both Nil and Extreme excision treatments, which were quite similar.

## Discussion

### Emergence traits

Partial cotyledon excision induced greater and more rapid seedling emergence in this experiment, consistent with some other reported results in the literature [8–10,36] but did not affect synchronicity of emergence compared with non-excised seedlings.

Emergence rate is influenced by various factors. Although emergence is positively correlated with oak acorn mass [19,37], there are inconsistent results in the literature in relation to partial excision effects on emergence. Higher total emergence and emergence rates were observed in partially excised acorns of *Q*. *variabilis* (in the present study) and in *Q*. *robur* acorns with a container cultivation regime [9]. However, in both Andersson and Frost’s study on *Q*. *robur* [13] and Mancilla-Leytón et al., with *Q*. *suber* [11], partial cotyledon excision did not promote emergence rate, and other studies reported that cotyledon excision reduced emergence rates of *Q*. *variabilis* and *Q*. *mongolica*, respectively [8,10]. These contrasting results may depend on whether acorns were container-grown or outplanted, or else directly sown in the field. Compared with direct sowing in the field, container-grown acorn emergence may be more affected by inhibitory substances stored in the cotyledon than by acorn nutrient reserves due to the high directed velocity of inhibitory substances and limited requirements for nutrients during seedling emergence (only less than 15% of the total acorn nutrient content) under nursery conditions. In addition, our results indicated that different intensities of excision affected emergence rate. The highest emergence rates occurred in Intermediate excision rather than Slight or Extreme excision treatments. We cannot say if this result applies to species other than *Q*. *variabilis*, because other studies did not compare emergence rates among different excision intensities [9–11].

Seedling emergence synchrony is often associated with environmental factors such as light, humidity, and temperature. It can be altered by various seed treatments, which include chilling, gibberellic acid, and priming with polyethylene glycol solutions [38,39]. Cotyledon excision is one such seed treatment, however in this study excised acorns emerged with similar synchronicity between different excision treatments. This suggests that the effect of acorn excision on emergence synchrony was relatively weak, compared with the effect of the uniform nursery surrounding conditions (water temperature and light) created by irrigation and the random positional switching of experimental trays.

For both total emergence and emergence rate, Intermediate excision had the highest values compared with Slight and Extreme excision treatments, which were significantly higher than Nil excision. Intermediate excision is therefore recommended for rapid and complete emergence.

### Nursery performance

Branco and colleagues [6] reported that seedling growth was positively correlated with seed reserves and nutrient contents, and this was also confirmed to be true in oak species [40]. Cotyledon excision can reduce acorn reserves and therefore negatively affect seedling growth [32,41–43]. In contrast, Giertych and Suszka [9] reported that slight levels of cotyledon loss did not suppress seedling growth but instead had an advantageous impact.

Our results and those of Giertych and Suszka [9] in their study of *Q*. *robur*, suggest that cotyledon excision does not always negatively affect the growth of *Q*. *variabilis* seedlings into the mature phase. In our study, effects on seedling sturdiness, dry mass, root morphology and nutrient concentration were generally not large with Slight or Intermediate excision. In addition, effects of cotyledon excision on *Q*. *variabilis* seedling nursery attributes varied with nursery fertilization.

#### Nursery seedling sturdiness and dry mass

Root collar diameter and Height/Diameter ratio are important sturdiness measurements. In this study, effects of cotyledon excision on *Q*. *variabilis* seedling sturdiness were quite small and generally non-significant. However, excision effects on nursery seedling dry mass were more complex. In other studies, slight excision (1/5-1/3) of acorns did not significantly affect seedling dry mass [9–11]. In contrast, in our study dry mass of *Q*. *variabilis* seedlings was generally reduced with increasing severity of excision treatments across both fertilizer treatments. Nursery fertilization somewhat increased seedling dry mass but did not markedly change the trend of decreasing dry mass with increasing severity of excision.

#### Root morphology

There is a lack of information about seedling root morphology in previous studies for acorn damage. As the taproot surface area accounted for more than 61% of total root area in this study (S3 Table), total root surface area represented the growth status of taproots to a certain extent despite not providing taproot surface area. Our results indicated that cotyledon excision negatively affected total root surface area as well as taproot growth in terms of dry mass and surface area. The first order lateral root growth was also reduced by excision. This may due to the immediate effects of cotyledon excision on root development in the early stages of seedling growth.

In contrast to excision, nursery fertilization promoted growth of both taproots and first-order lateral roots. This can be explained by the beneficial effect of nursery fertilization on root growth [44].

#### Nutrient concentrations

Giertych and Suszka [9] showed that after cutting off the distal end of cotyledons, nutrient concentrations of the young seedlings were not affected at the end of the growing season, and changes in the concentration of nutrients observed during growth were connected with physiological senescence. Our study results were generally in accordance with these findings. Although there were some significant effects of excision on seedling nutrient levels, most effects were not significant and there was no obvious pattern to the effects on N, P and K for the different organs.

In contrast, effect of nursery fertilization on seedling nutrient concentrations varied with elements (N, P or K) and organs (stem or root) while interacting with cotyledon excision. Overall, nursery fertilization led to higher seedling nutrient concentration, as demonstrated by Mancilla-Leytón and others on *Q*. *suber* [11].

Because metabolic resources stored in the large cotyledons are considered to be advantageous to seedling growth [45], reduced seedling growth with excision is currently explained by the excised-cotyledon loss perspective [6,40]. Mancilla-Leytón et al. [11] reported that when seedlings from extremely excised acorns were supplemented with fertilization, they exhibited similar height, root length and dry mass compared to the seedlings from intact acorns. However, growth reduction for Extreme treatments in the present study indicated that the reserve loss due to initial cotyledon excision cannot be fully compensated by subsequent fertilization. For instance, when two thirds of cotyledon were excised, average losses of N, P and K were 15.8 mg, 2.1 mg and 19.2 mg while fertilizer additions were 100 mg, 26 mg and 33 mg, respectively. Nutrient losses from excision were relatively small compared to the amount provided by nursery fertilization. This effect may be linked to the insufficient nutrient uptake capacity of roots for Extreme treatments. Most assuredly, initial growth of roots for oak seedlings were closely dependent on cotyledon nutrients. Excessive excision of cotyledons may restrain the growth of fine roots which play an important role in the subsequent process of nutrient uptake, as well as in seedling growth (for both stem and root) [46]. Accordingly the restrained development of the fine root system may have tended to decrease nutrient uptake capacity of roots for supplemental nutrients. On another hand, unequally distributed metabolic compounds in the cotyledon may play an important part in seedling growth as well as nutrient uptake for different cotyledon excision treatments. Steele and others [4] have identified that the content of tannin differed significantly between apical and distal parts of acorns. Cotyledon excision may have modified the magnitudes of both nutrient reserves and metabolic compounds. Overall, despite lacking direct evidence, the reduced growth performance of fertilized seedlings with increasing excision severity, suggests that nutrient reserve loss alone cannot completely explain the effects caused by cotyledon excision on seedling morphology and growth.

### Outplanting performance

Although seed germination and seedling growth of direct-sown *Q*. *variabilis* with cotyledon excision have been discussed [8,10], there is still a lack of information about outplanting performance of nursery seedlings for *Q*. *variabilis* as well as other oak species. Seedling outplanting performance is often closely linked to the size and nutrient concentration of nursery seedlings [47], which is consistent with the results of seedling measurements for High NF treatments in this study.

Though nursery fertilization cannot completely remedy adverse effects of excision treatments on nursery seedling sturdiness, biomass and nutrient concentration, it did promote dry mass increment for excised seedlings after outplanting. Unexpectedly, when seedlings from excised acorns were fertilized, Slight-Intermediate excision consistently promoted seedling dry mass increment after outplanting. In contrast, excision had no or even a negative effect on dry mass at the nursery. Contrasting effects between nursery and outplanting cannot be simply explained in terms of effects of excision and fertilization at the nursery phase but may be connected with the dynamics of seedling nutrient status and field growth environment. According to Villar-Salvador and colleagues [48], field growth of oaks was positively related to root growth capacity and plant nutrient concentrations (especially N concentration), which changed with seedling growth stages and was closely related to seedling size, as modified by field site conditions. Seedlings which had their cotyledon partially excised may have invested more in lateral and fine root growth (so that they could take up the nutrients of the fertilizers), thus they could accumulate more dry mass due to a better developed root system. Out of the three excision treatments, we found that the survival and growth of outplanted seedlings was most severely reduced by Extreme excision. Therefore, Extreme excision should be excluded as a seed treatment in *Q*. *variabilis* seedling cultivation and forest restoration projects.

## Conclusions

The influence of cotyledon excision on *Quercus variabilis* seedling cultivation is two-sided. It may have some disadvantages such as additional labor requirements and some reduction of seedling growth and root development in the nursery compared with seedlings from non-excised acorns. However, adverse effects on seedling growth and root development were not sustained into the second (field) growing season.

On the other hand, cotyledon excision will result in increased seedling emergence and therefore more efficient seedling production in terms of container preparation and seed sowing. It will also accelerate emergence speed although it will not result in more uniform time of emergence. The Intermediate excision treatment had the best emergence characteristics. More importantly, Intermediate acorn excision resulted in the highest seedling dry mass increment after the first outplanting season, which will benefit seedling survival and growth in subsequent seasons. Thus, Intermediate acorn excision before sowing is recommended for *Q*. *variabilis* seedling cultivation and forest restoration efforts. The excised part of the acorn would not necessarily be wasted, as acorns have many uses as a biomaterial for organic fertilizer production, animal feed, or starch production. In this way we can take full advantage of the excised parts of acorns and avoid wastage.

The current study also shows that cotyledon excision should not be considered separately from nursery fertilization and/or nursery soil nutrient status. For seedlings treated with cotyledon excision, restrained root system development tended to be improved by nursery fertilization although the effects of nutrient loss due to acorn consumption may not have been fully compensated. Nursery fertilization also has the ability to improve *Q*. *variabilis* seedling morphological growth in nursery and dry mass net increment after outplanting. Overall, in terms of nursery stock production and forest regeneration efficiency, Intermediate cotyledon excision can be a useful technique in nursery cultivation when combined with a reasonable level of nursery fertilization.

## Supporting information

S1 Fig The mean absolute emergence rate (first derivative for richards growth function, % new seedlings day-1) of *Quercus variabilis* seedlings. This figure corresponds to Fig 2, but intuitively showed the rapid emergence period of *Q*. *variabilis*.
(PDF)

S1 Table Soil conditions of the outplanting site for field research. Five points within the experiment site were sampled for physical traits and nutrients determining.
(PDF)

S2 Table Initial data for the amount of seedling emergence. Thirty acorns with different intensities of excision were sown in each try and a total of 450 acorns were initially employed for each excision treatment. This table corresponds to Fig 2A, but partitioned into replications.
(PDF)

S3 Table The mean root surface area (cm^2^) for different root lasses and the mean proportion (%) of taproot to total root surface area. (PDF)

## References

[1] Servello FA, Kirkpatrick RL. Regional variation in the nutritional ecology of ruffed grouse. J Wildlife Manage. 1987;51: 749–770. 10.2307/3801737

[2] Miyaki M, Kikuzawa K. Dispersal of *Quercus mongolica* acorns in a broad-leaved deciduous forest. Forest Ecol and Manage. 1988;25: 1–8. 10.1016/0378-1127(88)90129-6

[3] Harper JL, Moore KG. The shapes and sizes of seeds. Annual Review of Ecology & Systematics. 1970;1: 327–356. Available from: http://www.jstor.org/stable/2096777

[4] Steele MA, Knowles T, Bridle K, Simms EL. Tannins and partial consumption of acorns implications for dispersal of oaks by seed predators. Am Midl Nat. 1993;130: 229–238. 10.2307/2426123

[5] Mellanby K. The effect of some mammals and birds on the regeneration of oaks. J Appl Ecol. 1968;5: 359–366. 10.2307/2401566

[6] Branco M, Branco C, Merouani H, Almeida MH. Germination success, survival and seedling vigour of *Quercus suber* acorns in relation to insect damage. Forest Ecol Manage. 2002;166: 159–164. 10.1016/S0378-1127(01)00669-7

[7] den Ouden J, Jansen PA, Smit R. Jays, mice and oaks: predation and dispersal of *Quercus robur* and *Q*. *petraea* in north-western Europe In: Forget PM, editors. Seed Fate: Predation, dispersal and seedling establishment. Wallingford: CAB International; 2005 pp. 223–240.

[8] Hou XG, Yi XF, Yang YQ, Liu WJ. Acorn germination and seedling survival of *Q*. *variabilis*: effects of cotyledon excision. Ann. For. Sci. 2010;67: 711 10.1051/forest/2010036

[9] Giertych MJ, Suszka J. Consequences of cutting off distal ends of cotyledons of *Quercus robur* acorns before sowing. Ann For Sci. 2011;68: 433–442. 10.1007/s13595-011-0038-6

[10] Yang YQ, Yi XF. Partial acorn consumption by small rodents: implication for regeneration of white oak, *Quercus mongolica*. Plant Ecol. 2012;213: 197–205. 10.1007/s11258-011-0016-y

[11] Mancilla-Leytón JM, Cambrollé J, Figueroa ME, Vicente ÁM. Growth and survival of cork oak (*Quercus suber*) seedlings after simulated partial cotyledon consumption under different soil nutrient contents. Plant Soil. 2013;370: 381–392. 10.1007/s11104-013-1646-8

[12] Sonesson LK. Growth and survival after cotyledon removal in *Quercus robur* seedlings, grown in different natural soil types. Oikos. 1994;69: 65–70. 10.2307/3545284

[13] Andersson C, Frost I. Growth of *Quercus robur* seedlings after experimental grazing and cotyledon removal. Acta Bot Neerl. 1996;45: 85–94. 10.1111/j.1438-8677.1996.tb00497.x

[14] Herrera CM. Seed dispersal by vertebrates In: Herrera CM, editors. Plant animal interactions: An evolutionary approach. 1st ed UK: Blackwells Science; 2002 pp.185.

[15] Jiang MM, Cao L, Xiao ZS, Guo C. Frugivorous birds and its impacts on seed dispersal of wild cherry (*Prunus pseudocerasus*) in a Dujiangyan Forest, China. Chin J Zool. 2010;45: 27–34.

[16] Yu F, Niu KK, Jiao GQ, Lü HQ, Yi XF. Effect of small rodents on seed dispersal of five tree species in Xiaoxing’an Mountains. Journal of Northeast Forestry University. 2011;39: 11–13.

[17] Doody CN, O’Reilly C. Drying and soaking pretreatments affect germination in pedunculate oak. Ann. For. Sci. 2008;65: 509 10.1051/forest:2008027

[18] McCreary DD. The effects of stock type and radicle pruning on blue oak morphology and field performance. Ann. Sci. For. 1996;53: 641–648. 10.1051/forest:19960242

[19] Tilki F, Alptekin CU. Germination and seedling growth of *Quercus vulcanica*: effects of stratification, desiccation, radicle pruning, and season of sowing. New Forest. 2006;32: 243–251. 10.1007/s11056-006-9001-z

[20] Suszka B, Muller C, Bonnet-Masimbert M. Nasiona Drzew Leśnych. Odzbioru do siewu (Seeds of forest broadleaves from harvest to sowing). Warszawa—Poznań: PWN; 2000. pp. 307.

[21] Suszka B. Generative propagation In: Bugała W, editors. Dęby (*Quercus robur* L.; *Q*. *petraea* (Matt.) Liebl.)—Nasze drzewa leśne. Poznań: Bogucki Wydawnictwo Naukowe; 2006 pp. 305–388.

[22] Olson DF. Quercus L. In: Schopmeyer CS, editors. Seeds of woody plants in the United States. Washington DC, USA, USDA For. Serv: Agriculture Handbook; 1974. pp. 692–703.

[23] Bonner FT, Vozzo JA. Seed biology and technology of Quercus. Gen. Tech. Rep. SO-66. New Orleans, LA: U.S. Dept of Agriculture, Forest Service, Southern Forest Experiment Station; 1987. pp. 21. http://www.treesearch.fs.fed.us/pubs/810

[24] ISTA. Proceedings of the International Seed Testing Association. International rules for seed testing. Seed Sci Technol. 1999;27: 1–333.

[25] Finch-Savage WE, Farrant JM. The development of desiccation-sensitive seeds in *Quercus robur* L: Reserve accumulation and plant growth regulators. Seed Sci Res. 1997;7: 35–39. 10.1017/S0960258500003342

[26] Prewein C, Endemann M, Reinohl V, Salaj J, Sunderlikova V, Wilhelm E. Physiological and morphological characteristics during development of pedunculate oak (*Quercus robur* L.) zygotic embryos. Trees-Struct Funct. 2006;20: 53–60. 10.1007/s00468-005-0012-8

[27] Finch-Savage WE, Clay HA. Water relations of germination in the recalcitrant seeds of *Quercus robur* L. Seed Sci Res. 1994;4: 315–322. 10.1017/S096025850000235X

[28] Rasaneh Y, Kahnamooi MHM, Salehi P. Northern Iran forests qualitative and quantitative investigation. The Proceeding of Northern Iran Forests Management and Sustainable Development. 2001;1: 56–82.

[29] Grossman BC, Gold MA, Dey DC. Restoration of hard mast species for wildlife in Missouri using precocious flowering oak in the Missouri River floodplain, USA. Agroforest Syst. 2003;59: 3–10. 10.1023/A:1026124717097

[30] Garcíia-Cebriáan F, Esteso-Martinez J, Gil-Pelegrin E. Influence of cotyledon removal on early seedling growth in *Quercus robur* L.. Ann For Sci. 2003;60: 69–73. 10.1051/forest:2002075

[31] Hanley ME, Fegan EL. Timing of cotyledon damage affects growth and flowering in mature plants. Plant Cell Environ. 2007;30: 812–819. 10.1111/j.1365-3040.2007.01671.x 17547653

[32] Zhang WH, Duan BL, Zhou JY, Liu XJ. Water relations and activity of cell defense enzymes to water stress in seeding leaves of different provences of *Quercus variabilis*. Acta Phytoecologica Sinica. 2004;28: 483–490.

[33] Fu R, Guo SJ, Ma LY. The morphology and physiological response of mycorrhizal seedlings of *Quercus variabilis* under different soil moisture levels. Journal of Northwest Forestry University. 2011;26: 101–104.

[34] Li GL, Zhu Y, Liu Y, Wang JX, Liu JJ, Dumroese RK. Combined effects of pre-hardening and fall fertilization on nitrogen translocation and storage in *Quercus variabilis* seedlings. Eur J Forest Res. 2014;133: 983–992. 10.1007/s10342-014-0816-4

[35] Hay FR, Mead A, Bloomberg M. Modelling seed germination in response to continuous variables: use and limitations of probit analysis and alternative approaches. Seed Sci. Res. 2014;24:165–186. 10.1017/S096025851400021X

[36] Vallejo-Marin M, Dominguez CA, Dirzo R. Simulated seed predation reveals a variety of germination responses of neotropical rain forest species. Am J Bot. 2006;93: 369–376. 10.3732/ajb.93.3.369 21646197

[37] Gómez JM. Bigger is not always better: conflicting selective pressures on seed size in *Quercus ilex*. Evolution. 2004;58: 71–80. 10.1554/02-617 15058720

[38] Mohapatra SC, Arcila J, Johnson WH, Nelson LA. Induction of tobacco seed germination synchrony through dark preincubation. Agron J. 1987;79: 468–472. 10.2134/agronj1987.00021962007900030012x

[39] Pill WG, Kilian EA. Germination and emergence of Parsley in response to osmotic or matric seed priming and treatment with gibberellin. HortScience. 2000;35: 907–909.

[40] Merouani H, Branco C, Almeida MH, Pereira JS. Effects of acorn storage duration and parental tree on emergence and physiological status of cork oak (*Quercus suber* L.) seedlings. Ann For Sci. 2001;58: 543–554.

[41] Armstrong DP, Westoby M. Seedlings from large seeds tolerated defoliation better: a test using phylogenetically independent contrasts. Ecology. 1993;74: 1092–1100. 10.2307/1940479

[42] Hanley ME, Fenner M, Whibley H, Darvill B. Early plant growth: identifying the end point of the seedling phase. New Phytol. 2004;163: 61–66. 10.1111/j.1469-8137.2004.01094.x 33873793

[43] Hanley ME, May OC. Cotyledon damage at the seedling stage affects growth and flowering potential in mature plants. New Phytol. 2005;169: 243–250. 10.1111/j.1469-8137.2005.01578.x 16411928

[44] van den Driessche R. Effects of nitrogen and phosphorus fertilization on Douglas-fir nursery growth and survival after outplanting. Can J Forest Res. 2005;10: 65–70.

[45] Garcíia-Cebriáan F, Esteso-Martinez J, Gil-Pelegrin E. Influence of cotyledon removal on early seedling growth in *Quercus robur* L.. Ann For Sci. 2003;60: 69–73. 10.1051/forest:2002075

[46] Johnson PS, Shifley SR, Rogers R. The ecology and silviculture of oaks, 2nd ed MPG Books Group, Bodmin, UK; 2009 pp. 92–102.

[47] Villar-Salvador P, Puértolas J, Cuesta B, Peñuelas JL, Uscola M, Heredia-Guerrero N, et al Increase in size and nitrogen concentration enhances seedling survival in Mediterranean plantations. Insights from an ecophysiological conceptual model of plant survival. New Forests. 2012;43: 755–770. 10.1007/s11056-012-9328-6

[48] Villar-Salvador P, Peñuelas JL, Nicolás-Peragón JL, Benito LF, Domínguez-Lerna S. Is nitrogen fertilization in the nursery a suitable tool for enhancing the performance of Mediterranean oak plantations. New Forests. 2013;44: 733–751. 10.1007/s11056-013-9374-8

