# Supplementary material for: Combined effects of cotyledon excision and nursery fertilization on root growth, nutrient status and outplanting performance of Quercus variabilis container seedlings
Source: PLoS One. 2017 May 18;12(5):e0177002. doi: 10.1371/journal.pone.0177002 (PMC5436658; doi:10.1371/journal.pone.0177002)

S1 Fig. The mean absolute emergence rate (first derivative for Richards growth function, % new seedlings day^{-1}) of *Quercus variabilis* seedlings. This figure corresponds to Fig 2, but intuitively showed the rapid emergence period of *Q. variabilis.*

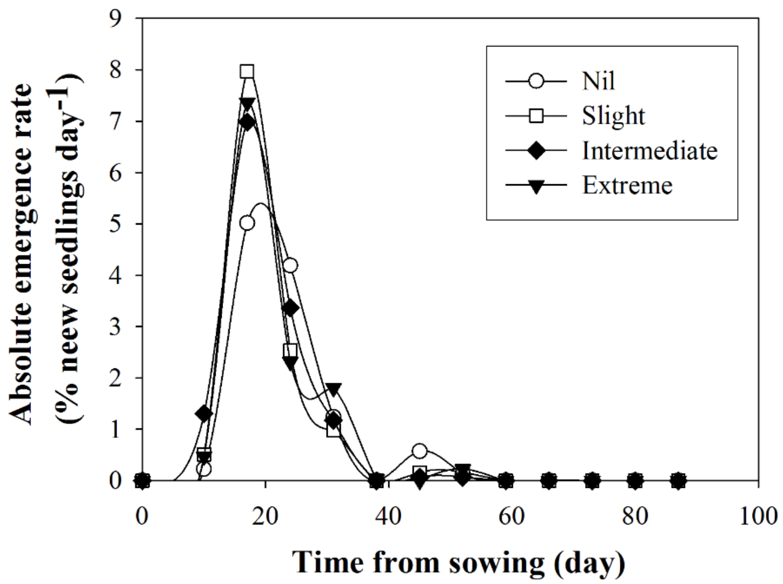

Supplement: S1 Fig — This figure corresponds to Fig 2, but intuitively showed the rapid emergence period of Q. variabilis. (PDF) [file pone.0177002.s001.pdf]
