# Supplementary material for: Combined effects of cotyledon excision and nursery fertilization on root growth, nutrient status and outplanting performance of Quercus variabilis container seedlings
Source: PLoS One. 2017 May 18;12(5):e0177002. doi: 10.1371/journal.pone.0177002 (PMC5436658; doi:10.1371/journal.pone.0177002)
Supplement: S1 Table — Five points within the experiment site were sampled for physical traits and nutrients determining. (PDF) [file pone.0177002.s002.pdf]

S1 Table. Soil conditions of the outplanting site for field research. Five points within the experiment site were sampled for physical traits and nutrients determining.

Indicators	Sample point [a]									
	Point 1		Point 2		Point 3		Point 4		Point 5	
	Top	Lower	Top	Lower	Top	Lower	Top	Lower	Top	Lower
**Soil particle composition** [b]	**Top**: 2-0.2 mm: 39.7%<br>0.2-0.05 mm: 15.7%<br>0.05-0.002 mm: 10.8%<br><0.002 mm: 15.7%					**Lower**: 2-0.2 mm: 76.4%<br>0.2-0.05 mm: 4.9%<br>0.05-0.002 mm: 9.9%<br><0.002 mm: 8.9%				
**pH**	6.19	7.16	6.04	6.68	6.28	6.94	6.15	6.80	6.16	6.89
**Organic carbon content (%)**	0.76	0.40	0.72	0.32	0.60	0.28	0.84	0.44	0.73	0.36
**Total N content (mg/kg)**	613.37	385.00	670.60	374.45	481.08	333.33	702.80	389.82	616.96	370.63
**Available P content (mg/kg)**	134.91	127.63	144.93	134.64	118.72	126.53	149.17	167.01	136.93	138.80
**Available K content (mg/kg)**	103.78	79.72	113.14	75.30	95.88	61.86	132.56	101.74	111.34	79.66

[a] Five sample points was "S" distributed within the experiment site. The depth of soil varied between 45 and 60 cm. We sampled two layers (Top and Lower) at each point as the root length for our experimental seedlings were less than 40 cm. The top layer was defined to 0~20 cm, and the lower layer 20~40 cm.

[b] The soil particle composition was determined with a mixture sample for topsoil and lower-layer soil, respectively.
