# Supplementary material for: Combined effects of cotyledon excision and nursery fertilization on root growth, nutrient status and outplanting performance of Quercus variabilis container seedlings
Source: PLoS One. 2017 May 18;12(5):e0177002. doi: 10.1371/journal.pone.0177002 (PMC5436658; doi:10.1371/journal.pone.0177002)
Supplement: S2 Table — Thirty acorns with different intensities of excision were sown in each try and a total of 450 acorns were initially employed for each excision treatment. This table corresponds to Fig 2A, but partitioned into replications. (PDF) [file pone.0177002.s003.pdf]

S2 Table. Initial data for the amount of seedling emergence. Thirty acorns with different intensities of excision were sown in each try and a total of 450 acorns were initially employed for each excision treatment. This table corresponds to Fig 2A, but partitioned into replications.

Excision treatments	Trays	Time schedule for seedling emergence recording									total
		2.26./0 d	2.29./3 d	3.6./10d	3.13./17d	3.20./24d	3.27./31d	4.2./38d	4.9./45d	4.16./52d	
**Nil**	1	Sowing	0	0	10	8	3	0	2	0	23
**Nil**	2	Sowing	0	0	10	9	3	0	1	0	23
**Nil**	3	Sowing	0	1	13	9	2	0	2	0	27
**Nil**	4	Sowing	0	0	10	9	3	0	0	0	22
**Nil**	5	Sowing	0	1	10	9	2	0	1	1	24
**Nil**	6	Sowing	0	0	10	9	3	0	1	0	23
**Nil**	7	Sowing	0	1	13	9	3	0	0	0	26
**Nil**	8	Sowing	0	1	10	9	2	0	1	1	24
**Nil**	9	Sowing	0	0	10	9	3	0	1	0	23
**Nil**	10	Sowing	0	0	10	7	3	0	2	1	23
**Nil**	11	Sowing	0	1	10	9	2	0	1	0	23
**Nil**	12	Sowing	0	1	12	9	3	0	0	1	26
**Nil**	13	Sowing	0	0	10	9	3	0	3	0	25
**Nil**	14	Sowing	0	1	10	9	2	0	2	0	24
**Nil**	15	Sowing	0	0	10	9	2	0	1	0	22
**Nil**	**total**	**/**	**0**	**7**	**158**	**132**	**39**	**0**	**18**	**4**	**358**
**Slight**	1	Sowing	0	0	13	5	1	0	0	0	19
**Slight**	2	Sowing	0	0	17	5	2	0	1	0	25
**Slight**	3	Sowing	0	1	15	6	2	0	0	0	24
**Slight**	4	Sowing	0	1	17	6	2	2	1	0	29

**Slight**	5	Sowing	0	1	16	5	0	0	2	0	24
**Slight**	6	Sowing	0	2	17	8	2	1	0	0	30
**Slight**	7	Sowing	0	1	14	6	4	0	1	0	26
**Slight**	8	Sowing	0	2	17	5	2	1	0	0	27
**Slight**	9	Sowing	0	1	17	6	2	0	0	0	26
**Slight**	10	Sowing	0	1	15	6	6	0	0	0	28
**Slight**	11	Sowing	0	2	19	3	2	1	0	0	27
**Slight**	12	Sowing	0	1	18	5	3	0	0	0	27
**Slight**	13	Sowing	0	2	17	5	1	0	0	0	25
**Slight**	14	Sowing	0	0	20	4	0	0	0	0	24
**Slight**	15	Sowing	0	1	17	5	2	0	0	0	25
**Slight**	**total**	**/**	**0**	**16**	**249**	**80**	**31**	**5**	**5**	**0**	**386**
**Intermediate**	1	Sowing	0	2	14	7	4	0	0	0	27
**Intermediate**	2	Sowing	0	3	14	6	3	0	0	0	26
**Intermediate**	3	Sowing	0	5	15	7	2	0	0	0	29
**Intermediate**	4	Sowing	0	3	14	8	0	1	0	0	26
**Intermediate**	5	Sowing	0	1	17	7	3	0	0	0	28
**Intermediate**	6	Sowing	0	0	14	7	2	0	0	0	23
**Intermediate**	7	Sowing	0	3	15	5	2	0	1	0	26
**Intermediate**	8	Sowing	0	4	16	7	1	1	0	0	29
**Intermediate**	9	Sowing	0	2	14	8	3	0	0	0	27
**Intermediate**	10	Sowing	0	3	17	7	2	0	0	0	29
**Intermediate**	11	Sowing	0	4	14	9	3	0	0	0	30
**Intermediate**	12	Sowing	0	2	13	8	2	0	0	0	25
**Intermediate**	13	Sowing	0	4	16	7	2	0	1	0	30

**Intermediate**	14	Sowing	0	3	13	6	5	0	0	0	27
**Intermediate**	15	Sowing	0	2	14	7	3	0	0	0	26
**Intermediate**	**total**	**/**	**0**	**41**	**220**	**106**	**37**	**2**	**2**	**0**	**408**
**Extreme**	1	Sowing	0	2	12	4	4	0	0	0	22
**Extreme**	2	Sowing	0	1	18	5	3	0	1	0	28
**Extreme**	3	Sowing	0	1	16	2	5	1	0	0	25
**Extreme**	4	Sowing	0	1	12	5	4	0	1	0	23
**Extreme**	5	Sowing	0	0	17	5	4	0	0	0	26
**Extreme**	6	Sowing	0	1	17	4	5	0	0	0	27
**Extreme**	7	Sowing	0	1	13	5	4	0	0	0	23
**Extreme**	8	Sowing	0	3	11	5	4	0	1	0	24
**Extreme**	9	Sowing	0	1	14	6	2	0	0	0	23
**Extreme**	10	Sowing	0	0	17	4	5	0	1	0	27
**Extreme**	11	Sowing	0	1	18	5	4	0	1	0	29
**Extreme**	12	Sowing	0	0	16	5	4	0	0	0	25
**Extreme**	13	Sowing	0	1	17	7	3	0	1	0	29
**Extreme**	14	Sowing	0	2	17	5	4	0	0	0	28
**Extreme**	15	Sowing	0	0	17	6	2	0	1	0	26
**Extreme**	**total**	**/**	**0**	**15**	**232**	**73**	**57**	**1**	**7**	**0**	**385**
