# Supplementary material for: Combined effects of cotyledon excision and nursery fertilization on root growth, nutrient status and outplanting performance of Quercus variabilis container seedlings
Source: PLoS One. 2017 May 18;12(5):e0177002. doi: 10.1371/journal.pone.0177002 (PMC5436658; doi:10.1371/journal.pone.0177002)
Supplement: S3 Table — (PDF) [file pone.0177002.s004.pdf]

S3 Table. The mean root surface area (cm^2) for different root lasses and the mean proportion (%) of taproot to total root surface area.

Cotyledon excision	Nursery fertilization	Lateral and fibrous root surface area (cm^2)	Taproot surface area (cm^2)	Total root surface area (cm^2)	Proportion of taproot (%)
**Nil**	**Low NF**	28.38	55.56	83.94	66
**Slight**	**Low NF**	37.39	58.40	95.79	61
**Intermediate**	**Low NF**	32.83	58.79	91.62	64
**Extreme**	**Low NF**	23.23	48.55	71.78	68
**Nil**	**High NF**	43.42	77.98	121.40	64
**Slight**	**High NF**	30.01	51.14	81.15	63
**Intermediate**	**High NF**	34.41	64.59	99.00	65
**Extreme**	**High NF**	30.83	60.90	91.73	66
